# Surface plasmon resonance, molecular docking, and molecular dynamics simulation studies of lysozyme interaction with tannic acid

**DOI:** 10.1002/fsn3.4315

**Published:** 2024-07-22

**Authors:** Emir Alper Türkoğlu, Ilgaz Taştekil, Pemra Özbek Sarica

**Affiliations:** ^1^ Department of Pharmaceutical Biotechnology, Faculty of Pharmacy University of Health Sciences Turkey İstanbul Türkiye; ^2^ Department of Bioengineering Institute of Pure and Applied Sciences, Marmara University İstanbul Türkiye; ^3^ Department of Bioengineering, Faculty of Engineering Marmara University İstanbul Türkiye

**Keywords:** chicken egg white lysozyme, molecular docking, molecular dynamics, surface plasmon resonance, tannic acid

## Abstract

Lysozyme (L_ZM_) is an important enzyme in medicine and industry. Tannic acid (TA) is used in brewing, wine industry, and as a food flavor enhancer. In nutritional and food science, L_ZM_ interacts with TA, notably in wine and saliva. This study aimed to investigate the binding interaction between L_ZM_ and TA using surface plasmon resonance, molecular docking, and molecular dynamics simulation. Chicken egg white lysozyme (CEWL_ZM_) was applied as a model protein. Tri‐N‐acetylchitotriose (NAG_3_), the known inhibitor of CEWL_ZM_, was used in the redocking experiments to determine the precise binding location within the complex. The average binding energies obtained from docking NAG_3_ and tannic acid to the target structure of CEWL_ZM_ were found to be −6.46 ± 0.05 kcal/mol and −7.52 ± 0.39 kcal/mol, respectively. The binding free energy of the CEWL_ZM_‐TA complex was then calculated as −27.61 kcal/mol by MMPBSA based on MD simulation trajectories. The observed interactions between the ligands and the lysozyme structure were mainly driven by hydrophobic, van der Waals, and H‐bond interactions formed by the active site residues. MD simulations showed consistent and satisfactory binding distances between CEWL_ZM_ and TA throughout the analysis. SPR analysis was performed using 1X PBS buffer (pH 7.4) as coupling and running buffers, 30 μL/min as flow rate, and 2.5 mg/mL CEWL_ZM_. Serial concentrations of TA (20–150 μM) were injected through immobilized CEWL_ZM_, and the *K*_*D*_ value of CEWL_ZM_‐TA binding was obtained as 4.17 × 10^−5^ M. This study could enhance existing literature and pave the way for future research in food science and oral biology.

## Graphical abstract

The interaction between chicken egg white lysozyme (CEWL_ZM_) and tannic acid (TA) was investigated using computational techniques and SPR binding analysis. The computational results showed average binding energies and minimum distance values for the CEWL_ZM_‐TA interaction and the number of H‐bond formations in this complex. For the experimental study, SPR analysis was used to determine the K_D_ value of CEWL_ZM_‐TA binding. This study could enhance existing literature and pave the way for future research in food science and oral biology.

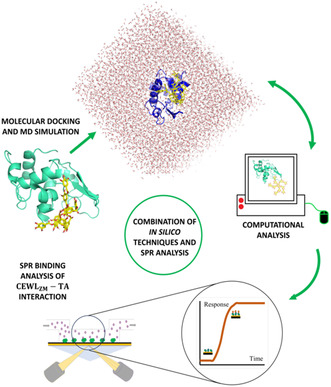

## 1 INTRODUCTION

Lysozyme (L_ZM_) is a hydrolytic enzyme (EC 3.2.1.17) found in various body secretions and fluids of living organisms. Its catalytic properties make it a valuable component in pharmaceutical products and the food industry, where it facilitates the hydrolysis of β‐1,4‐glycosidic linkages between N‐acetylglucosamine and N‐acetylmuramic acid within the peptidoglycan structure of bacterial cell walls (Liburdi et al., 2014; Niyonsaba & Ogawa, 2005). Additionally, this enzyme plays a critical role in oral biology, functioning as a salivary defense protein (Sun et al., 2016). Given its extensive use in both biological and industrial contexts, it is crucial to monitor the bioactivity and stability of L_ZM_ due to its interactions with other molecules, involving electrostatic, hydrogen, and hydrophobic forces (Su et al., 2019).

L_ZM_ can be categorized into various types, including invertebrate (i‐type), goose (g‐type), chicken (c‐type), plant, bacterial, and phage types. Human lysozyme (hL_ZM_) is specifically classified as a c‐type lysozyme. Chicken egg white lysozyme (CEWL_ZM_), with a molecular mass of 14.4 kDa and a p*I* of 10.9 (Jalili‐Firoozinezhad et al., 2020), shares 59% sequence homology with hL_ZM_ (Ferraboschi et al., 2021). It consists of two domains and 129 amino acid residues (Abeyrathne et al., 2014; Cheetham et al., 1992; Pechkova et al., 2010). Domain 1 (D1) consists of residues 40 to 88, while domain 2 (D2) consists of residues 1–39 and 89–129 (Young et al., 1994). The active site cleft is located between these two domains, and involves the main active site residues Glu35, Asp52, Leu56‐Asn59, Arg61‐Trp63, Arg73, Ile98, Asp101, Asn103, Ala107, and Trp108 (Cheetham et al., 1992). Among these, the role of Glu35 and Asp52 is noteworthy since they play an essential role in catalytic processes such as the cleavage of the bacterial cell wall components (Held & van Smaalen, 2014; Phillips, 1966).

Tannins, which are chemically diverse, are found in numerous plant families throughout the world. They are found in high concentrations in almost every part of the plant, including bark, wood, leaves, fruits, roots, and seeds (Khanbabaee & van Ree, 2001). Tannins have the ability to interact with proteins (Zhang et al., 2023), but their size plays a critical role in this process. For efficient cross‐linking, the tannin molecule should be large enough to bind to protein chains at multiple sites but small enough to penetrate protein fibers (Chung et al., 1998). There are several types of interactions involved in protein‐tannin complexes, such as hydrogen bonding, hydrophobic interaction, electrostatic attraction, and covalent bonding by oxidation. The binding of tannins to proteins primarily occurs through multiple hydrogen bonds formed between the phenolic hydroxyl groups of tannins and the carboxyl groups of proteins (Quan et al., 2019). Tannic acid (TA), as a hydrolysable tannin, has diverse applications in several industries. It is used as an additive in medical products (Khan et al., 2000), as a clarifying agent in the brewing and wine industries, and as a flavoring agent in various food products (Singh & Kumar, 2020) due to its typical phenolic characteristics (Nie et al., 2017). TA, especially in wine and tea, can lead to the inhibition of L_ZM_ and/or alteration of enzyme bioactivity (Guzzo et al., 2011); moreover, it also acts to induce astringency. This condition is associated with the formation of insoluble precipitates due to hydrogen bonding and/or hydrophobic interactions between the phenolic hydroxyl groups of TA and proteins present in saliva. In the development of biopharmaceutical formulations, this astringency mechanism has been used to design a simple and sustained‐release system for L_ZM_ by mixing the enzyme with TA, known as the TANNylation process (Utatsu et al., 2023).

To date, many investigations have been conducted on the binding of exogenous compounds with medically important proteins (Chen et al., 2022;   Wang, Wu, et al., 2019) due to their potential effects on many biochemical and physiological processes in living bodies (Wang, Wang, et al., 2019;   Zhao et al., 2022). However, a limited number of studies have explored the L_ZM_‐TA interaction within the realms of food science and oral biology. Various techniques, including Fourier‐transform infrared (FT‐IR), fluorescence, dynamic light scattering, and circular dichroism (CD) spectroscopy, have been employed in these investigations. Nevertheless, these techniques have predominantly depicted the TA‐L_ZM_ complex (Li et al., 2020; Su et al., 2019) without providing insights into the binding affinity between the two molecules. Surface plasmon resonance (SPR) is an optical phenomenon that allows real‐time and label‐free quantification of molecular interactions (Gade et al., 2022) with high sensitivity and specificity, requiring a small volume of sample for analysis (Çimen et al., 2022; Ertürk et al., 2016). SPR has gained widespread popularity in the assessment of molecular interactions between ligands and analytes, providing valuable biophysical data such as kinetics, affinity, and thermodynamics (Gaudreault et al., 2022). Due to its remarkable properties, this technology has emerged as a pivotal tool in various fields, including diagnosis, cellular monitoring, genotyping, food testing, environmental monitoring, and the analysis of biomolecular binding interactions (Mariani & Minunni, 2014). SPR systems are extensively configured as prism coupling (prism/metal layer/analyte), waveguide coupling, fiber coupling, and grating coupling, depending on the sensing structure. The prism‐based (also known as Kretchmann's configuration) has been a widely used type among them (Velasco‐Garcia, 2009). The configuration of the gold surface on a prism is commonly manufactured for sensing platforms due to its several advantages, such as high chemical stability, durability, and low oxidizing power (Kumar et al., 2022). Detectable signals are generated from this metal layer due to binding interactions that cause refractive index changes (Stewart et al., 2008).

The aim of the study is to reveal the nature of the interaction between tannic acid and lysozyme using in silico and in vitro experiments. The study provides the first description of the binding interactions of L_ZM_ with TA using SPR spectroscopy. In addition to the experimental SPR kinetic analysis, the structural features and dynamics of the L_ZM_‐TA were also investigated by in silico techniques, including molecular docking of the L_ZM_ and TA and further molecular dynamics simulation analysis to understand the mechanism underlying the binding of two molecules. For this purpose, CEWL_ZM_ was chosen as a model protein.

In summary, the scientific activities carried out in this study were: (i) prediction of the 3D structure of the CEWL_ZM_‐TA complex, (ii) description of the actual binding sites of CEWL_ZM_ with TA and demonstration of the amino acids involved in this interaction using molecular docking studies and MD simulation analysis, (iii) preparation of the self‐assembled monolayer (SAM) in order to immobilize the CEWL_ZM_ before kinetic analysis studies, (iv) serial injection of different concentrations of TA through the immobilized CEWL_ZM_ for kinetic analysis and finally (v) obtaining the association (*k*_*a*_), dissociation (*k*_*d*_) rate constants, and equilibrium constant (*K*_*D*_) from the kinetics of the binding curves.

## 2 MATERIALS AND METHODS

### 2.1 Reagents and materials

CEWL_ZM_, 11‐mercaptoundecanoic acid (MUA), N‐ethyl‐N′‐(3‐(dimethylamino)propyl) carbodiimide (EDC), N‐hydroxysuccinimide (NHS), ethanolamine hydrochloride (EA‐HCl), KH_2_PO_4_, Na_2_HPO_4_, NaOH, and hydrogen peroxide (H_2_O_2_) were commercially obtained from Sigma‐Aldrich (Saint Louis, MO, USA). TA was supplied by Alfa Aesar (Alfa Aesar Company, USA). KCl and NaCl were purchased from Carlo Erba (Milan, Italy). Polyethersulfone (PES) syringe filter with 0.22 μm pore size, sulfuric acid (H_2_SO_4_), and ethanol were commercially obtained from Isolab (Turkey). Disposable 20 mL BD plastic syringes with Luer‐Lock fittings were utilized in microfluidic applications. The bare gold SPR sensor chip and matching fluid were purchased from Biosensing Instrument Inc. (Tempe, AZ, USA).

### 2.2 Instruments

The reagents employed in this study, including CEWL_ZM_, TA, H_2_O_2_, and stock solutions, were stored at 4°C. EDC was preserved directly at −20°C. All the water utilized in buffer preparation and kinetic studies was purified through a Direct Q^®^3 UV water purification system (Millipore Corp., France). Prior to use, laboratory glassware was submerged overnight in a 5% nitric acid solution, then rinsed with ultrapure water, and dried in a dust‐free environment. Accurate liquid transfer and sampling were conducted utilizing single‐channel pipettes (Eppendorf Research Plus, Eppendorf AG, Germany). The weighing of all reagents and chemicals was performed with an analytical balance (Ohaus PA224C, Ohaus Corp., USA). The pH values of the solutions were measured by a benchtop pH meter (Seven Compact, Mettler Toledo, Switzerland). An ultrasonic bath (Elmasonic S 60 H, Elma Schmidbauer GmbH, Germany) was used for homogenization and degassing of the solutions.

In this study, binding of CEWL_ZM_‐TA interactions was conducted by a BI‐4500A SPR instrument (Biosensing Instrument Inc., Tempe, AZ, USA), which employs p‐polarized laser light (*λ* = 670 nm) to analyze binding kinetics. The SPR instrument incorporates several key features, including precise sample delivery with minimal dispersion to allow for rapid kinetic measurements and the ability to produce high‐quality data that can distinguish actual bindings from various secondary effects. Data S1 presents the enhanced SPR data quality procedures. Different units are used to quantify SPR signals, such as refractive index unit (RIU), resonance unit (RU), and mDeg (Charania et al., 2013). In this study, the unit of SPR signal was established as RU, where a change of 0.0001° in resonance angle corresponds to 1 RU (Park et al., 2012).

### 2.3 Computational docking studies

Prior to docking TA to CEWL_ZM_, redocking of a known ligand (NAG_3_) to CEWL_ZM_ was conducted to ensure the correct docking conditions. The ligand‐bound complex (CEWL_ZM_‐NAG_3_) structure 1HEW (with a resolution of 1.75 Å and an *R*‐value of 0.229) (Cheetham et al., 1992) was used as a reference for the redocking process. Autodock Tools (ADT) (Morris et al., 2009) was employed to generate structure files, while Autodock Vina (Eberhardt et al., 2021; Trott & Olson, 2010) was used for the docking process. Hydrogen atoms were assigned to the polar residues of CEWL_ZM_, Kollman charges were added, and the protein structure was checked for missing atoms. The crystallized NAG_3_ was obtained from the main pdb file (1HEW). The hydrogen atoms were added to the ligand. After adding the hydrogen atoms, energy minimization was applied by the Universal Force Field (UFF) to avoid any steric clashes and maintain the geometric stabilization of the ligand (Dallakyan & Olson, 2015). After geometrical optimization, the NAG_3_ ligand was converted to a pdbqt file through ADT, which automatically added Gasteiger charges to it. Using ADT, a grid‐box was generated based on the NAG_3_‐binding site of the complex, and five individual docking runs were processed for redocking.

Once the validated docking conditions were established as a result of the redocking process (a grid‐box with a size of 25 × 25 × 25 Å and an exhaustiveness value of 8), the same procedure was applied to dock TA. TA, which is a relatively large ligand with a molecular weight of 1701.2 g/mol, was retrieved from the PubChem Database (Kim et al., 2021).

### 2.4 Molecular dynamics (MD) simulations

The simulation was conducted in a cubic box solvated with TIP3P water, and the system was neutralized with the necessary amounts of sodium/chloride ions. MD simulations were performed using GROMACS (Abraham et al., 2015) and the CHARMM36‐all‐atom force field (Vanommeslaeghe et al., 2010). A number of water box models have been improved, including SPC and TIP3P, with slightly different characteristics to mimic the aqueous environment. They differ in the way they reproduce the enthalpy of evaporation and the density of water. These water box models have been studied with different force fields to match the model and force field used in the simulation and to optimize the force fields (Berensen et al., 1981; Jorgensen et al., 1983). In a previous study, the SPC model was found to give a more accurate result with the CHARMM all‐atom force field when only the protein is in its native form. However, TIP3P was found to be the most appropriate model for the mutant state of the protein (Nguyen et al., 2014). As the ligand‐bound proteins were identified in this study, the TIP3P water box model was used with the CHARMM36 all‐atom force field based on the literature.

Ligand parameters were generated using CGenFF and the cgenff_charmm2gmx_py3_nx1.py script (Soteras Gutiérrez et al., 2016; Vanommeslaeghe et al., 2010; Vanommeslaeghe & MacKerell, 2012; Yu et al., 2012). Energy minimization was performed to avoid any inappropriate geometric conditions and clashes. The simulation systems were equilibrated using NVT (constant number of particles, volume, and temperature) and NPT (constant number of particles, pressure, and temperature) ensembles. Particle pressure and temperature were managed using a Parrinello‐Rahman barostat and a Berendsen thermostat. To keep the particles within a cut‐off distance while taking into account an additional buffer distance, the Verlet cut‐off system was employed. System electrostatics were set based on Particle‐Mesh Ewald (PME) electrostatics. The same parameters were set for the energy minimization. MD simulations were then conducted for both ligand‐free and ligand‐bound CEWL_ZM_ at a pressure of 1 bar and a temperature of 310 K for 100 ns with the same thermostat and barostat conditions. Two parallel simulations were carried out for each condition.

### 2.5 Trajectory analysis

The Root Mean Square Deviation (RMSD) and Root Mean Square Fluctuation (RMSF) values were calculated based on the alpha carbons of the amino acids. The conformations of the trajectories were superimposed on the average conformation of each trajectory for the calculations. The minimum distances between the ligand and the amino acids of the protein were calculated to investigate the amino acid residues involved in the interaction. In addition, analysis was performed to investigate the number of H‐bonds between the ligand and the protein. Furthermore, the equilibrated trajectories were used for the following analysis, and the secondary structures of the ligand‐free and ligand‐bound proteins were calculated. The interactions formed between the protein and the ligand were classified using the ProLif library (Bouysset & Fiorucci, 2021), and Molecular Mechanics Poisson‐Boltzmann Surface Area (MMPBSA) analysis was then performed to calculate the binding free energy of the complex (Valdés‐Tresanco et al., 2021).

### 2.6 Preparation of SAM on SPR gold chip surface

Before the functionalization of the gold surface, the chip surface was cleaned with piranha solution [H_2_SO_4_/H_2_O_2_ (7:1 v/v)] for 20 s. Piranha‐cleaned gold surface was rinsed with pure ethanol and ultra‐pure quality water and then dried with nitrogen gas. After the surface cleaning steps, the gold chip was immersed in a 1 mM MUA solution for 24 h at 22°C to establish SAM on the gold surface. After SAM formation, the chip was washed three times with pure ethanol to remove unbounded MUA residues. The modified chip was then dried in dust‐free conditions and mounted onto the prism of the SPR system for surface activation and CEWL_ZM_ immobilization.

### 2.7 CEWL_ZM_ immobilization on a gold chip

The SPR system was rinsed with degassed 1X PBS (pH 7.4) before the experimental steps. The 1X PBS (pH 7.4) solution was injected into the system to achieve a stable baseline on the sensorgram before activation of MUA. To activate the MUA‐modified gold surface, a 1:1 (v/v) EDC‐NHS mixture, consisting of NHS at 0.05 M and EDC at 0.2 M, was injected into the system for 450 s at a flow rate of 40 μL/min. Subsequently, 2.5 mg/mL CEWL_ZM_ in PBS buffer (pH 7.4) was injected at a flow rate of 30 μL/min for 790 s after activation, with the reference channel left unexposed to CEWL_ZM_. Then non‐specific binding was blocked by injecting a 1.0 M EA‐HCl solution onto the surface for 300 s. PBS was used as the running and coupling buffer throughout the entire experimental procedure.

### 2.8 SPR kinetic analysis of TA interaction with CEWL_ZM_

Binding interactions between immobilized CEWL_ZM_‐TA were evaluated using a double referencing protocol, including blank injection and subtraction of the reference channel. The SPR sensor surface was fixed as 25°C. To determine the kinetic parameters, serial concentrations of TA solutions (20–150 μM) were injected into the channels at a flow rate of 30 μL/min for 360 s. After each injection, the surface was regenerated using a 100 mM NaOH solution at a flow rate of 10 μL/min for 1080 s. The binding of CEWL_ZM_ and TA resulted in an increase in the refractive index on the binding surface. The association rate constant (*k*_*a*_), dissociation rate constant (*k*_*d*_), and equilibrium constant (*K*_*D*_) were obtained by fitting the curves of binding kinetics. Binding kinetic parameters of the immobilized CEWL_ZM_‐TA interaction were calculated using the SPR Kinetic Analysis Software of Biosensing Instrument (version 2.0.0.3).

## 3 RESULTS AND DISCUSSION

### 3.1 L_ZM_ –TA interaction nature

Tannins are known as protein‐complexing agents and interact with L_ZM_ due to their complex‐forming behaviors in several artificial (Benucci et al., 2016; Tirelli & De Noni, 2007) and natural environments. Guzzo et al. (2011) showed that phenolic compounds in wine tend to bind salivary proteins such as L_ZM_ and decrease their bioactivity. The interaction between L_ZM_ and TA results in the formation of a substantial complex, where TA effectively shields the active sites of the enzyme, thereby obstructing the enzyme's interaction with its substrate (Figure S1). In this assembly, TA effectively binds the active site of L_ZM_ through robust non‐covalent interactions. There is a limited body of scientific literature addressing the L_ZM_‐TA interaction, emphasizing the complexity of their association and the impact on enzyme bioactivity (Su et al., 2019; Utatsu et al., 2023). These kinds of studies require considerable time, effort, and specific compounds (Soares et al., 2007) and do not serve to demonstrate the affinity between the two molecules. In this study, we conducted molecular docking and molecular dynamics simulations to uncover the structural characteristics of the potential interaction between CEWL_ZM_ and TA. Subsequently, an SPR study was performed to determine the kinetic and affinity constants of this interaction.

### 3.2 Computational molecular docking

**FIGURE 1:**
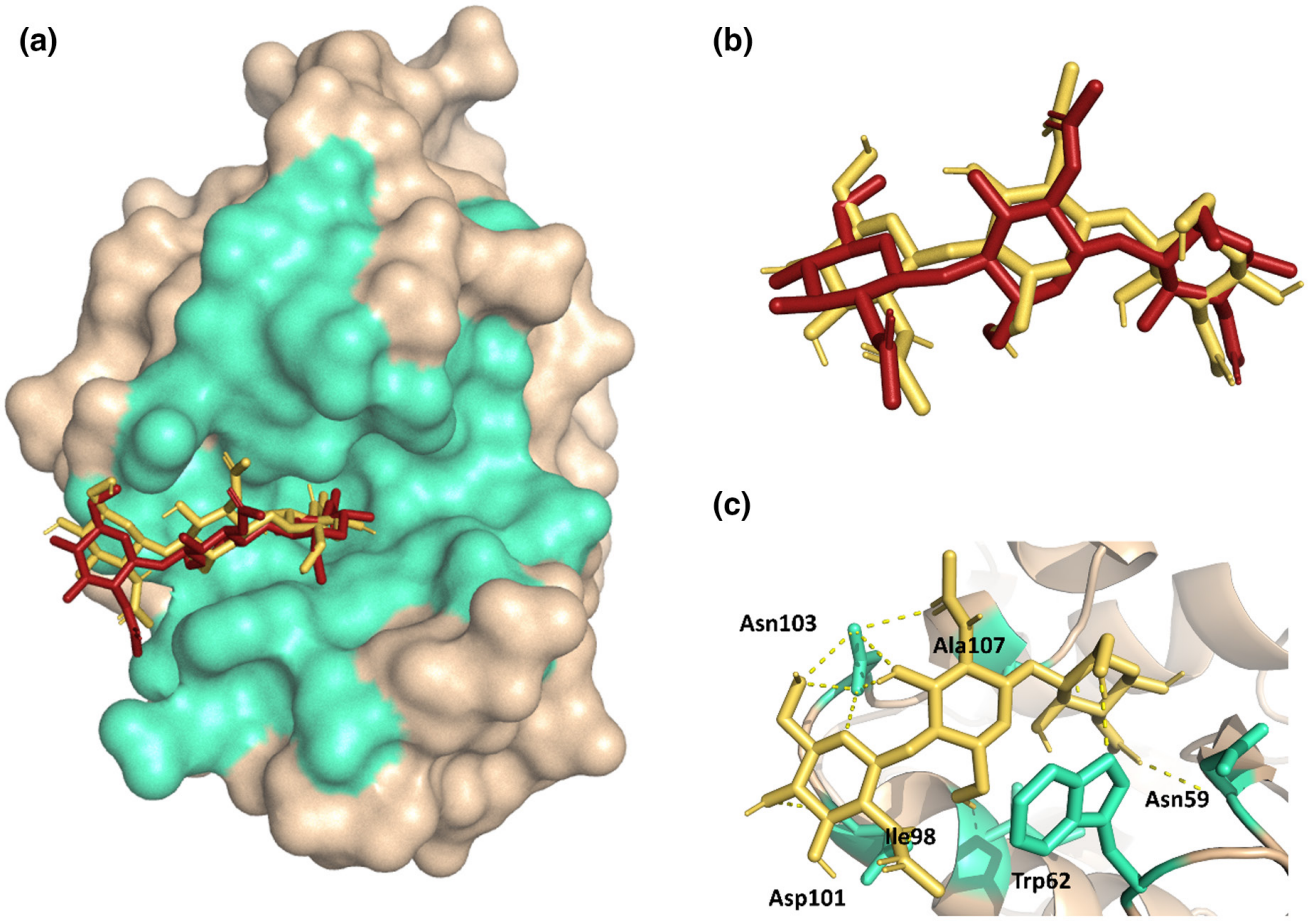
Redocking results: (a) Overlay of the redocked NAG_3_ and the crystal structure of CEWL_ZM_‐NAG_3_. The active site cleft is shown as green‐cyan and the rest of the protein is shown in wheat color. (b) Original and docked ligand conformations were aligned. A representative model was selected from five replicate experiments for display. (c) Polar interactions between CEWL_ZM_ and NAG_3_ are shown as an interaction network using Pymol (The PyMol Molecular Graphics System, 2020).

Using the 1HEW structure as a reference molecule, molecular redocking simulations were performed with a grid box size of 25 × 25 × 25 Å. As a result of redocking, experimentally verified ligand conformation was successfully obtained with the same active residues (Figure 1a–c). Hence, the molecular docking protocol developed in this study could be used in future parts of the study with confidence. Five separate runs were performed, and the resulting pose of a representative docking run was detailed in Figure 1 and Table 1 (all the other poses are detailed in Figures S2 and S3). The energy values obtained from five individual runs were −6.5, −6.5, −6.4, −6.4, and −6.5 kcal/mol, with an average of −6.46 ± 0.05 kcal/mol (Table 1). Hydrogen bond (H‐bond) formations with the active sites are maintained in all structures, as illustrated in Table S1, for the redocking process (Table S2, on the other hand, illustrates the H‐bond conformations obtained from the molecular docking process of TA to CEWL_ZM_). As a result of the redocking process, the correct binding pose was successfully obtained.

**TABLE 1:** Binding energies obtained from molecular docking of CEWL_ZM_‐NAG_3_ and CEWL_ZM_‐TA complexes. The binding energies of five individual runs for each complex were recorded with average (AVG) binding energies and standard deviations.

	1	2	3	4	5	AVG binding energy (kcal/mol)
Redocking of CEWL_ZM_‐NAG_3_ complex	−6.5	−6.5	−6.4	−6.4	−6.5	−6.46 ± 0.05
Docking of CEWL_ZM_‐TA complex	−7.9	−7.0	−7.3	−7.5	−7.9	−7.52 ± 0.39

### 3.3 Molecular docking of TA

**FIGURE 2:**
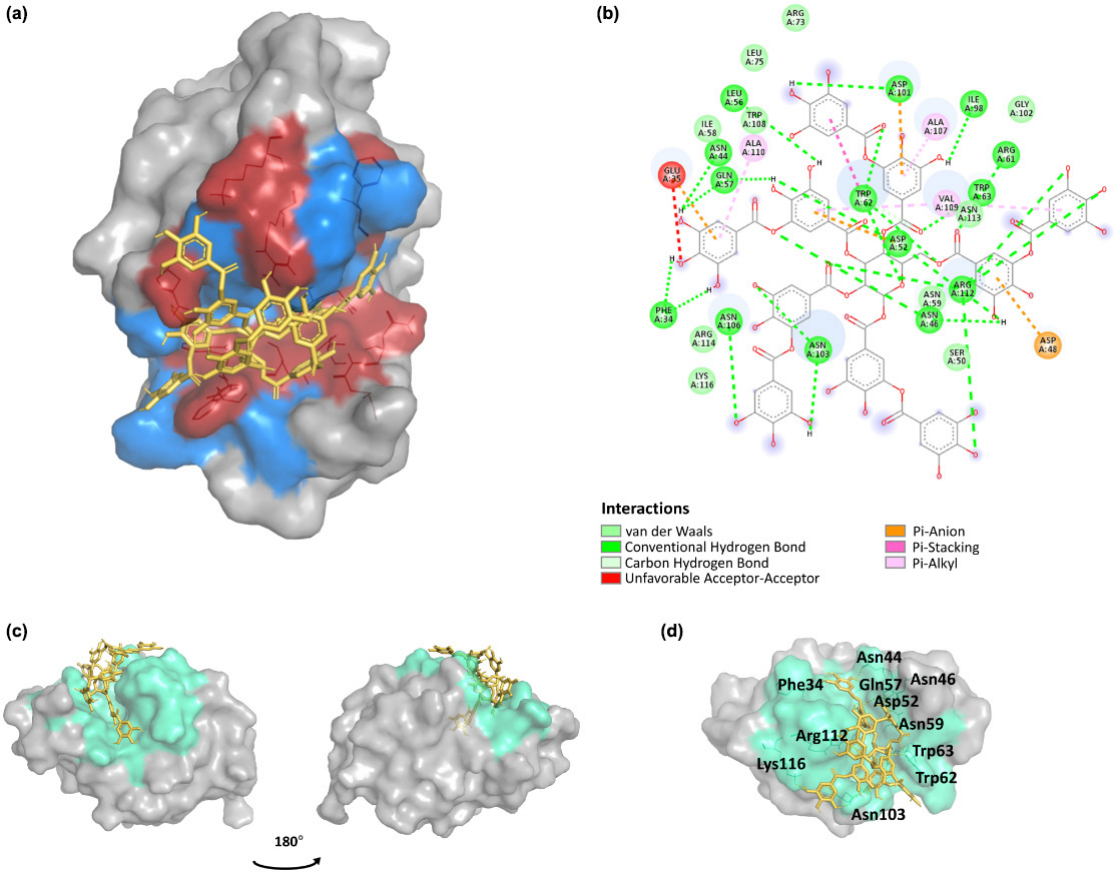
CEWL_ZM_‐TA complex obtained as a result of molecular docking: (a) Polarity of the active site is shown. The polar residues are displayed as red and the non‐polar residues are displayed as blue. The protein is shown in a surface representation and TA is shown in sticks. (b) 2D interaction map of the CEWL_ZM_‐TA complex (BIOVIA & Dassault Systèmes, 2020). (c) How TA occupies the active site cleft was shown from different perspectives (The PyMol Molecular Graphics System, 2020). (d) Polar contacts of TA on the binding region of the CEWL_ZM_ are labeled with a three‐letter code of amino acids.

Molecular docking of TA to CEWL_ZM_ was performed using the same conditions. The binding energies obtained from molecular dockings are given in Table 1, and the interacting amino acid residues are shown in Figures 2, S4, and S5.

TA interacts with CEWL_ZM_ through an intense network of van der Waals and electrostatic interactions and covalent H‐bonds (Figure 2a,b) in particular. The binding energies obtained from molecular dockings showed a satisfactory level of interaction between CEWL_ZM_ and TA (−7.52 ± 0.39 kcal/mol) compared to the interaction between CEWL_ZM_ and NAG_3_ (−6.46 ± 0.05 kcal/mol) (Table 1). According to the results, TA directly blocks the entrance of the active site crevice and tightly sits on the protein (Figure 2c), suggesting that CEWL_ZM_ and TA interact to form a tight network (Table S1). Both polar and non‐polar interactions between CEWL_ZM_ and TA were observed as a result of the molecular docking process.

### 3.4 Analysis of MD simulations

**FIGURE 3:**
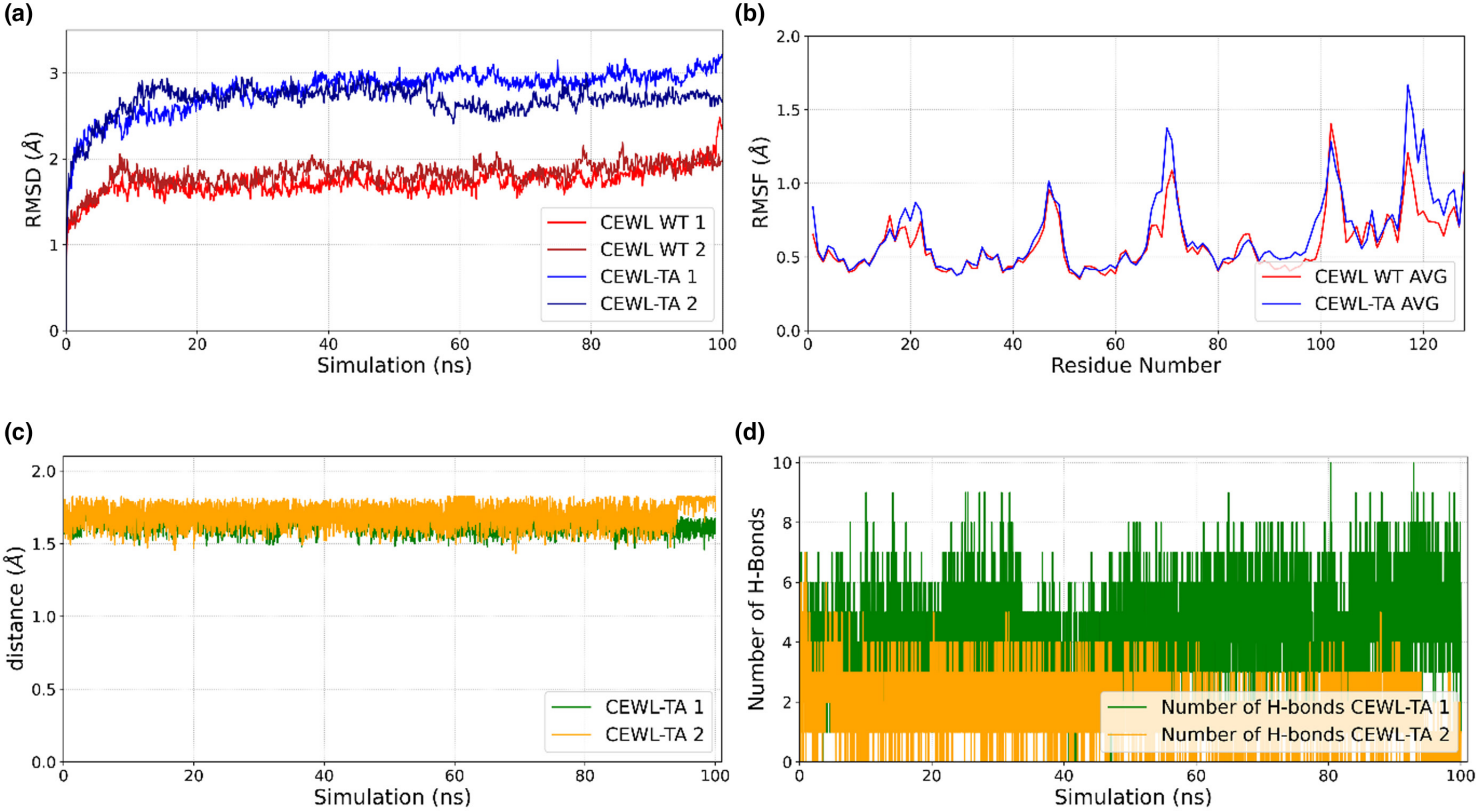
MD simulation analysis where two parallel runs were displayed for each simulation: (a) RMSD trajectory of unbound CEWL_ZM_ (red and dark red) and bound CEWL_ZM_‐TA complex (blue and dark blue). (b) The average RMSF values calculated based on the equilibrated part of the simulation. (c) Minimum distances between CEWL_ZM_ and the ligand TA observed throughout the simulations. (d) H‐bond interaction map of the complex structures throughout the 100 ns simulations.

Both the complex (Figure 3) and the ligand structures' (Figure S6) RMSD calculations based on C_α_ (Figure 3a) suggest the presence of a stable interaction between CEWL_ZM_ and TA throughout the simulations. For the calculation of RMSF (Figure 3b), the last 80 ns of the trajectories belonging to the equilibrium portion of the simulations were taken, and the average values are shown in Figure 3.

RMSF profiles (Figure 3b) indicate the flexibilities of the residues and can be informative in detecting the fluctuation of the residues upon binding. Significant fluctuations were observed for the residues Tyr20, Arg21, Arg68, Thr69, Pro70, Gly71, Ile98, Val99, Ser100, Asp101, Lys116, Gly117, Thr118, Asp119, Val120, and Gln121, which mostly reside in the loop regions and in the α_4_‐helix (Ser100 and Asp101). Among these, Ile98 and Asp101 are within the active site cleft.

The minimum distance values among CEWL_ZM_ and TA were in the range between 1.43 Å and 1.73 Å (Figure 3c) throughout the simulations, suggesting satisfying binding. The number of H‐bonds formed between CEWL_ZM_ and TA was also consistent with other trajectory analyses throughout the simulations (Figure 3d).

In contrast to molecular docking, enthalpy, and entropy of the system were also considered in the calculation of the binding energy. The binding free energy of the complex molecule was calculated as −27.61 kcal/mol from the MD trajectory by MMPBSA. During the simulations, the structural dynamics analysis between the active site residues of CEWL_ZM_ and TA revealed a dense network of hydrophobic interactions, van der Waals interactions, and H‐bond interactions. The intensity of these hydrophobic, van der Waals, and H‐bond interactions formed between the ligand and the protein explains the low binding energy value calculated by MMPBSA.

**FIGURE 4:**
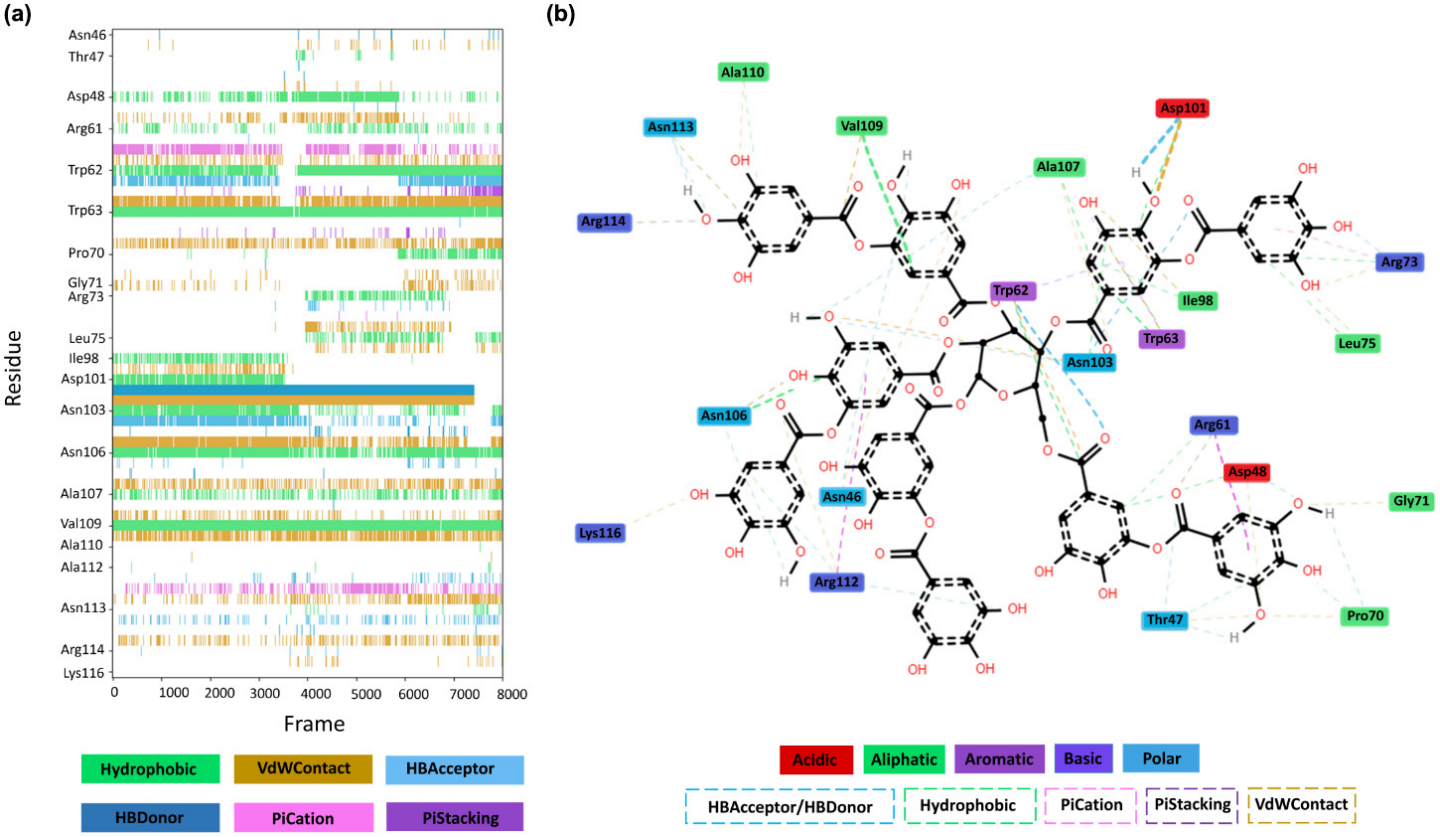
The interaction analysis of MD simulations: (a) The amino acid residues contributed to the interaction with TA throughout the simulation and the types of interaction. (b) 2D interaction map of MD simulation analysis. Figures were produced by the ProLIF library (Bouysset & Fiorucci, 2021).

The secondary structure profiles of the free and ligand‐bound proteins were calculated (Figure S7a,b). The presence of TA did not affect the secondary structure of the protein, and the secondary structure components remained same (Figure S7c). Further, interaction analysis was performed on the concatenated ligand‐bound trajectories to analyze the interactions occurring between CEWL_ZM_ and TA (Bouysset & Fiorucci, 2021). The residues most frequently interacting with the ligand were determined to be Val109 (99.9%), Trp63 (99.3%), Trp62 (93.8%), Asp101 (92.6%), Asn106 (90.9%), Arg61 (77.3%), Asn103 (75.5%), Arg112 (64.3%), Asp48 (52.6%), and Ala107 (51.1%), which are mainly the active site residues, throughout the simulation (Figure 4a). Interactions between these residues and the ligand were mostly driven by hydrophobic, van der Waals, and H‐bond donor interactions, although other types of interactions were also formed between the other residues and ligand (Figure 4a,b).

Although the studies related to the binding mechanism of the lysozyme–tannic acid complex are very limited, there are a few studies focusing on investigating the binding mechanism of lysozyme and TA, which were performed in vitro (Su et al., 2019; Tian et al., 2018). In each case, the binding occurs through intense intermolecular non‐covalent interactions and hydrophobic interactions, regardless of the temperature of the environment. However, *K*_*D*_ values were shown to be affected by temperature differences (Su et al., 2019; Tian et al., 2018). The computationally determined intense hydrophobic interaction network formed between TA and lysozyme and the experimentally determined *K*_*D*_ value are consistent with the literature.

Finally, the chirality of the protein in the presence of TA was evaluated with respect to the obtained computational data. Chirality in proteins results from the asymmetry of the amino acid sequence that comprises the protein. In our study, it was shown that the presence of TA did not affect the secondary structure of CEWL_ZM_. Although TA masked the entire active site cleft by binding, it is unlikely to alter the chirality of CEWL_ZM_ because significant changes did not occur in the global structure of the protein.

### 3.5 Design of SAM and CEWL_ZM_ immobilization on SPR gold sensor surface

**FIGURE 5:**
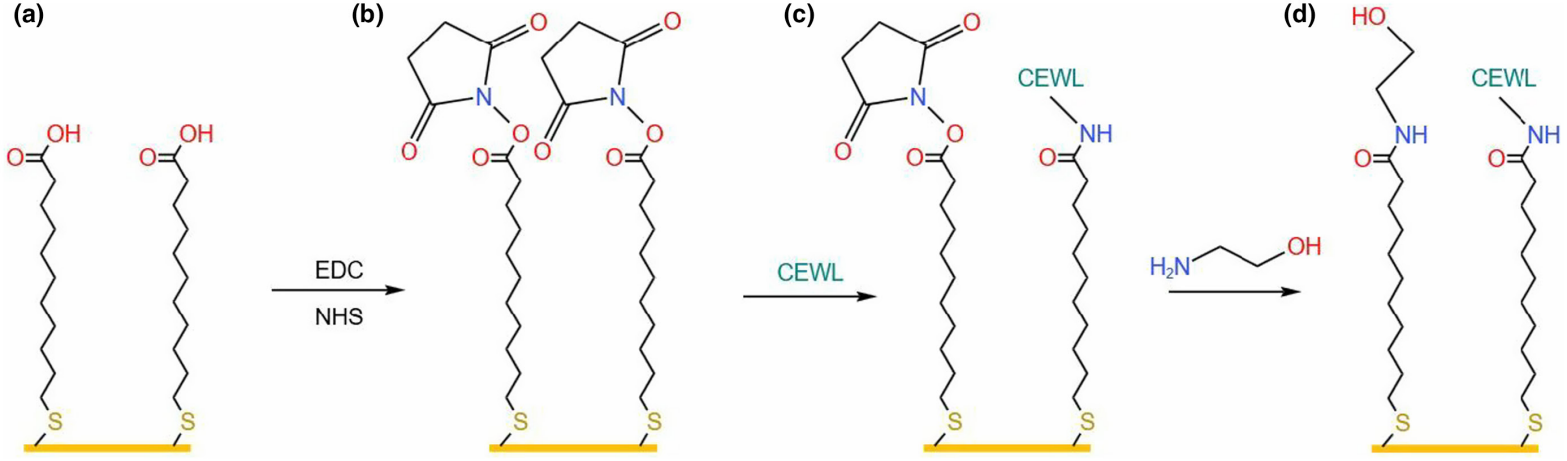
Schematic illustration of CEWL_ZM_ immobilization on MUA‐based SAM on Au surface: (a) MUA design on Au surface, (b) activation with NHS‐EDC solution, (c) CEWL_ZM_ immobilization, and (d) blocking of remaining activating groups. Modified from Xiao et al. (2011).

**FIGURE 6:**
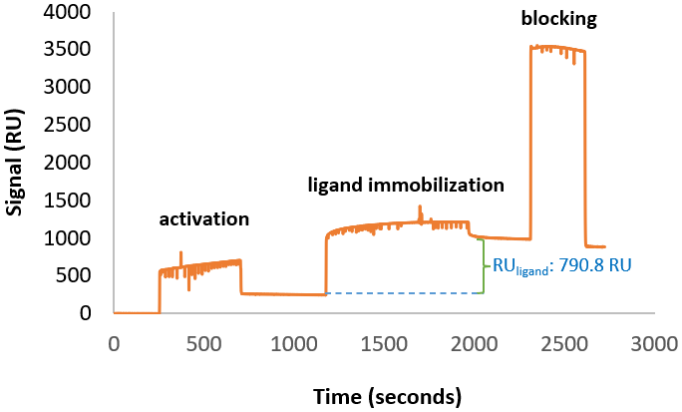
SPR sensorgram of CEWL_ZM_ immobilization step on functionalized gold surface.

An SPR analysis followed the computational applications to combine the structural features and the dynamics of the complex obtained from the computational analysis with the valuable kinetic data. MUA, a long‐chain alkanethiol, is frequently employed in the creation of SAMs for SPR binding analysis. It is a negatively charged chemical at neutral pH (Asphahani et al., 2011). In this study, 1 mM MUA was applied for the immobilization of CEWL_ZM_ on SAM. This functionalized sensor surface is widely used for amine coupling applications (Su et al., 2005). A freshly prepared activation solution (EDC‐NHS mixture) was injected through MUA‐based functionalized SAM on the gold sensor surface to activate the channels. In the activation step, a flow rate of 40 μL/min was preferred due to its less perturbation. After the activation step, CEWL_ZM_ was immobilized on the gold surface through electrostatic interaction (Steudle & Pleiss, 2011) and covalent amide formation. EA solution was injected into the SPR channels to block any remaining active binding sites (Figure 5). This step is also crucial for eliminating loosely bound ligand from the activated chip surface (D'aurelio et al., 2020). In the activation step, the SPR signal exhibited a sharp initial increase with the exposure of the activation solution to the sensor surface. This solution enhanced surface quality by activating MUA. Then, exposure to CEWL_ZM_ caused a substantial signal increase, which indicated successful CEWL_ZM_ immobilization (Figure 6) (Xiao et al., 2011).

In this study, one channel was used for ligand binding analysis, while the other channel served as a reference channel for reference subtraction in SPR binding analysis. The reference channel compensates for the bulk index of refractive shifts, non‐specific signals, and temperature drifts (Naimushin et al., 2002).

### 3.6 Analysis of SPR sensorgram data

There has been a growing interest in studying the interactions between L_ZM_ and exogenous substances (Asemi‐Esfahani et al., 2022; He et al., 2021). The techniques typically applied in these interactions do not provide real‐time monitoring of binding analysis. Up to this point, only a small number of studies have investigated the interaction between L_ZM_ and exogenous substances with the SPR technique. For instance, (i) SPR binding analysis was performed to obtain the affinity data of various nanobodies toward L_ZM_. The results indicate that these nanobodies exhibit strong affinity for immobilized L_ZM_ on the CM5 sensor surface with low *K*_*D*_ values in the range of 5–1300 nM (Birchenough et al., 2021). (ii) The binding kinetics between the surface protein antigen (pAc) and L_ZM_ were assessed through SPR analysis. In this investigation, pAc was immobilized onto the CM5 chip surface as the ligand, and three varying concentrations of L_ZM_ solutions were serially injected into the immobilized pAc. The resulting *K*_*a*_ and *K*_*d*_ values were calculated as 3.63 ± 0.03 × 10^3^ M^−1^ s^−1^ and 1.72 ± 0.04 × 10^−5^ s^−1^, respectively (Senpuku et al., 1996).

The binding of CEWL_ZM_ to TA has been studied using SPR analysis. In SPR binding studies, the maximum binding capacity of the sensor is essential for ideal SPR binding data. 2.5 mg/mL CEWL_ZM_ immobilization using 1X PBS (pH 7.4) as running and coupling buffer at 30 μL/min on the 1 mM MUA functionalized surface produced a useful theoretical maximum binding capacity of the surface (*R*_max_) (Türkoğlu, 2024). According to the SPR signal of CEWL_ZM_ immobilization at the end of the exposure step (RU_Ligand_ value: 790.8 RU in Figure 6), the *R*_max_ theoretical was calculated as 93.42 RU, which was useful in SPR binding analysis (Yoshitani et al., 2005). For basic optimization of the regeneration solution, 100 mM NaOH was injected at 10 μL/min to desorb TA from immobilized CEWL_ZM_ surfaces. According to our optimization studies for the regeneration step, (i) 50 mM and 75 mM NaOH solutions do not achieve effective regeneration at flow rates in the range of 10 and 60 μL/min and (ii) NaOH solutions with high molar concentrations do not allow meaningful SPR interaction studies. These situations can be explained as follows: (a) a low concentration of NaOH does not provide effective and complete removal of TA from the CEWL_ZM_ surface, (b) a high concentration of NaOH disrupts the structure of the immobilized CEWL_ZM_, and as a result, the disrupted protein surface is unable to interact effectively with TA.

**FIGURE 7:**
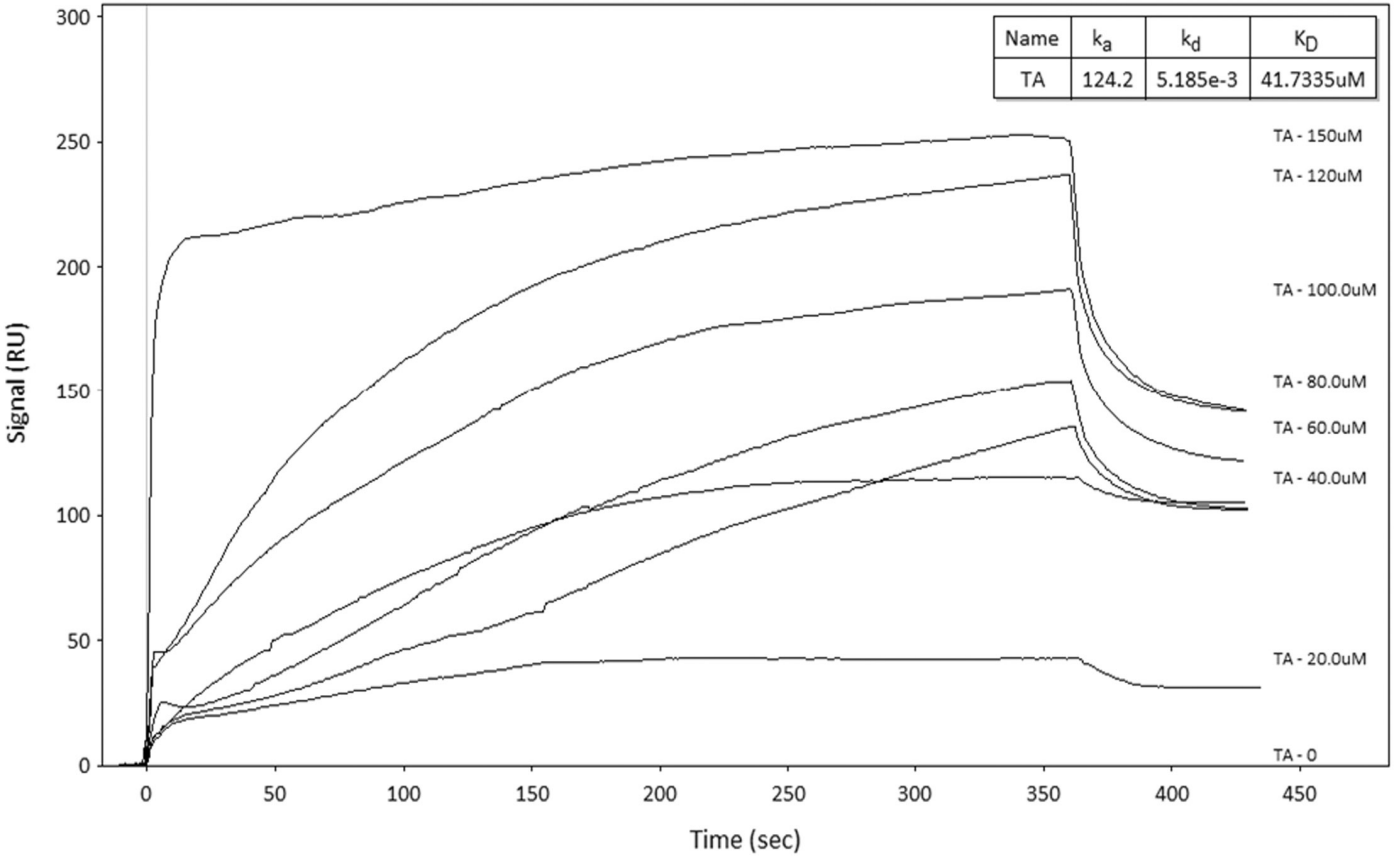
Experimental SPR binding curves of CEWL_ZM_ with TA at different concentrations (20–150 μM) (uM on the sensorgram produced by Kinetic Analysis Program refers to μM).

The association rate constant (*k*_*a*_) signifies how complex formations are generated per second, while the dissociation rate constant (*k*_*d*_) represents the fraction of complexes that decay per second. These critical parameters, *k*_*a*_ and *k*_*d*_, were determined through SPR binding studies. Finally, the equilibrium constant (*K*_*D*_), which illustrates the ligand's affinity for any analyte, was calculated (Sharifi et al., 2017). According to the results, *k*_*a*_, *k*_*d*_, and *K*_*D*_ were determined to be 124.2 M^−1^ s^−1^, 0.0051 s^−1^, and 4.17 × 10^−5^ M, respectively (Figure 7).

In brief, prior to SPR binding studies, molecular docking studies and molecular dynamics simulations were conducted to understand the binding mechanism of the CEWL_ZM_‐TA complex. The computational studies revealed the interaction network, including the amino acid residues involved in the interaction and the types of bonds formed between two molecules. The existence of non‐covalent interactions of the CEWL_ZM_‐TA complex was demonstrated by the redocking of a known lysozyme inhibitor, NAG_3_, to CEWL_ZM_ (PDB ID: 1HEW; Cheetham et al., 1992; Svobodová et al., 2010). After five separate run results, the average binding energies for CEWL_ZM_‐NAG_3_ and CEWL_ZM_‐TA were found to be −6.46 ± 0.05 kcal/mol and −7.52 ± 0.39 kcal/mol, respectively, with a stronger binding energy between CEWL_ZM_‐TA compared to that of CEWL_ZM_‐NAG_3_. The H‐bond and the minimum distance analysis calculated with respect to the MD simulations supported the strong and continuous interaction between CEWL_ZM_‐TA. In this manner, Svobodová et al. (2010) showed that the *K*_*D*_ value of the CEWL_ZM_‐NAG_3_ complex was found to be 39.0 ± 6.4 μM using an automated ESI‐MS chip configuration set‐up combined with a syringe pump. Jecklin et al. (2008) also demonstrated a comparison of three different electrospray ionization methods for the mass spectrometry (MS) detection of the non‐covalent CEWL_ZM_‐NAG_3_ complex. Among these techniques, chip‐based nanoESI was applied to MS to evaluate the performance of determining the *K*_*D*_ value of CEWL_ZM_ binding to NAG_3_. According to the results, the *K*_*D*_ value was obtained as 39.8 ± 8.8 μM. In our study, a *K*_*D*_ value in the same affinity range for the CEWL_ZM_‐TA interaction was determined to be 41.7 μM according to the SPR binding analysis result. The equilibrium dissociation constant (*K*_*D*_) for the CEWL_ZM_‐TA interaction closely matched the *K*_*D*_ value for the CEWL_ZM_‐NAG_3_ interaction (López‐Méndez et al., 2021; Zhang et al., 2016), indicating the presence of a relatively high affinity between CEWL_ZM_ and TA. The findings from our binding interaction analysis, as facilitated by SPR binding assays, have provided valuable insights into the CEWL_ZM_‐TA interaction and supported the reliability of the computational analysis. Nevertheless, the binding energy calculations can be conducted with the molecular mechanics Poisson–Boltzmann surface area (MMPBSA) analysis for further studies.

## 4 CONCLUSION

Here, the binding interaction between CEWL_ZM_ and TA was examined using a comprehensive approach consisting of in silico and in vitro techniques, including molecular docking, molecular dynamics simulations, and surface plasmon resonance. TA showed potential binding properties according to (i) in silico analysis due to the intensity of hydrophobic, van der Waals, and H‐bond interactions formed between the protein and the ligand and (ii) comparison of the average binding energy results of CEWL_ZM_‐TA and CEWL_ZM_‐NAG_3_. Further, the hydrophobic interaction network determined by MD simulation analysis is also consistent with the literature (Su et al., 2019; Tian et al., 2018). In addition to the computational structural data, the binding pattern analysis of CEWL_ZM_‐TA was then used to understand the kinetics of the structure using SPR technology. SPR serves as a versatile and reliable platform for real‐time monitoring of protein–compound binding, facilitating the determination of affinity constants in these ligand–analyte interactions. The *K*_*D*_ value of CEWL_ZM_‐TA is consistent with in silico investigations and supports studies conducted by different bio‐interaction analysis techniques in the literature. While further analysis is necessary to enhance our understanding of L_ZM_‐TA interactions, this study highlights the efficacy of SPR as a valuable real‐time monitoring technique for binding interaction analysis. In addition to our current focus on computational and analytical interaction techniques, we also look forward to exploring the impact of exogenous compounds on L_ZM_'s bioactivity in our future investigations.

## AUTHOR CONTRIBUTIONS

**Emir Alper Türkoğlu:** Conceptualization (lead); investigation (equal); methodology (equal); resources (lead); software (equal); validation (equal); visualization (equal); writing – original draft (equal); writing – review and editing (equal). **Ilgaz Taştekil:** Investigation (equal); methodology (equal); software (equal); validation (equal); visualization (lead); writing – original draft (equal). **Pemra Özbek Sarica:** Investigation (equal); methodology (equal); software (supporting); validation (lead); writing – original draft (equal); writing – review and editing (equal).

## FUNDING INFORMATION

This study was designed with the resources obtained from a Scientific Research Project Fund of the University of Health Sciences Turkey under project number 2020/032. The publication fee of the study was funded by TÜBİTAK (The Scientific and Technological Research Council of Türkiye).

## CONFLICT OF INTEREST STATEMENT

The authors declare that they have no conflict of interest.

## Supporting information


Data S1.

## Data Availability

The data that support the findings of this study are available on request from the corresponding author.
