# Supplementary material for: Surface plasmon resonance, molecular docking, and molecular dynamics simulation studies of lysozyme interaction with tannic acid
Source: Food Sci Nutr. 2024 Jul 22;12(10):7392–404. doi: 10.1002/fsn3.4315 (PMC11521726; doi:10.1002/fsn3.4315)
Supplement: Supplementary file 1 — Data S1. [file FSN3-12-7392-s001.docx]

Supporting Information

**Surface plasmon resonance, molecular docking and molecular dynamics simulation studies of lysozyme interaction with tannic acid**

Emir Alper TÜRKOĞLU^1*^, Ilgaz TAŞTEKİL^1,2^, Pemra ÖZBEK SARICA^3^,

^1^University of Health Sciences Turkey, Faculty of Pharmacy, Department of Pharmaceutical Biotechnology, İstanbul, Türkiye

^2^Marmara University, Institute of Pure and Applied Sciences, Department of Bioengineering, İstanbul, Türkiye

^3^Marmara University, Faculty of Engineering, Department of Bioengineering, İstanbul, Türkiye

Corresponding authors: alper.turkoglu@sbu.edu.tr

**Table of contents**

**Number of pages:** 15

**Note S1.** Procedures for enhancing SPR data quality.

**Figure S1.** 3D structure representation of chicken egg white lysozyme (CEWL_ZM_) (PDB ID: 1HEW) (Cheetham et al., 1992). D1 and D2 domains are shown in green and pink, respectively. The active site cleft of CEWL_ZM_ is shown in a sphere representation (wheat).

**Figure S2.** Superimposition of 5 runs, coloured as red, green, blue, yellow and magenta, respectively.

**Figure S3.** 2D interaction maps of the 5 redocking applications. a) 2D interaction map of the first redocking. b) 2D interaction map of the second redocking. c) 2D interaction map of the third redocking. d) 2D interaction map of the fourth redocking. e) 2D interaction map of the fifth redocking.

**Table S1.** Table of interactions between CEWL_ZM_ and NAG_3_.

**Table S2.** Table of interactions between CEWL_ZM_ and TA.

**Figure S4.** Superimposition of 5 docking runs of CEWL_ZM_-TA complex, colored as red, green, blue, yellow and magenta, respectively.

**Figure S5.** 2D interaction maps obtained from the molecular docking application for CEWL_ZM_-TA complex: a) Molecular docking run 1. b) Molecular docking run 2. c) Molecular docking run 3. d) Molecular docking run 4. e) Molecular docking run 5.

**Figure S6.** Ligand RMSD throughout the simulation.

**Figure S7.** The secondary structure analysis of free and ligand-bound CEWL_ZM_. The structure components are i) structure as seagreen (indicating the sum of α-helix + β-sheet + β-bridge + turn elements), ii) coil as yellow, iii) β-sheet as blue, iv) β-bridge as orange, v) bend as red, vi) turn as crimson, vii) α-helix as green, viii) 5-helix as purple and ix) 3-helix cyan. a) The secondary structure elements of the free CEWL_ZM_. b) The secondary structure of CEWL_ZM_ in the presence of TA. c) Comparison of the secondary structure of free CEWL_ZM_ and CEWL_ZM_-TA complex.

**Note S1.** Procedures for enhancing SPR data quality.

SPR chips may exhibit minor performance variations. Hence sensor fine tune was carried out at the beginning of each experiment to achieve highest quality binding data. The presence of air bubbles in solutions is a common challenge in the preparation of buffers and SPR system operation (Katsamba et al., 2002; Rich et al., 2008). To effectively eliminate and reduce the air bubbles (i) in the solutions, sonication method was utilized to degas of the solution because of the efficient air bubble removal characteristic of the method (Cho et al., 2021). Besides (ii) for the trapping air bubbles in flow systems, chip and gasket surfaces were cleaned and checked for any deformations, cracks, burns …etc. Subsequently, mounting steps for chip and gasket were properly applied. Finally, debubble action of the system was performed through the flow system.

In the preparation of binding kinetic investigations, the microfluidic flow system was thoroughly rinsed with 20 mM NaOH for obtaining optimal quality of data. Basic maintenance and valve cleaning procedures of SPR system were periodically performed according to the maintenance procedures of the instrument.


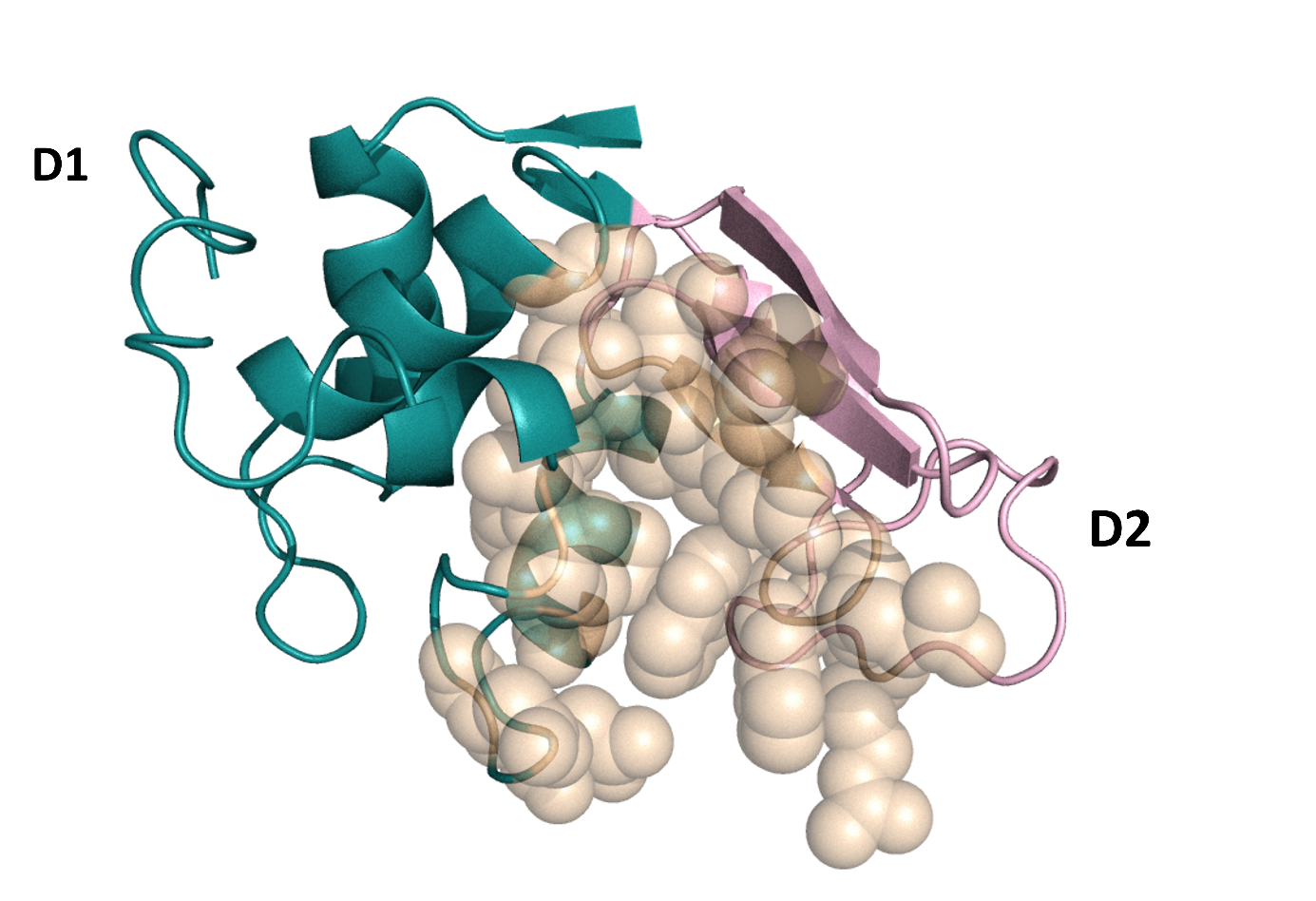


**Figure S1.** 3D structure representation of chicken egg white lysozyme (CEWL_ZM_) (PDB ID: 1HEW) (Cheetham et al., 1992). D1 and D2 domains are shown in green and pink, respectively. The active site cleft of CEWL_ZM_ is shown in a sphere representation (wheat).

**
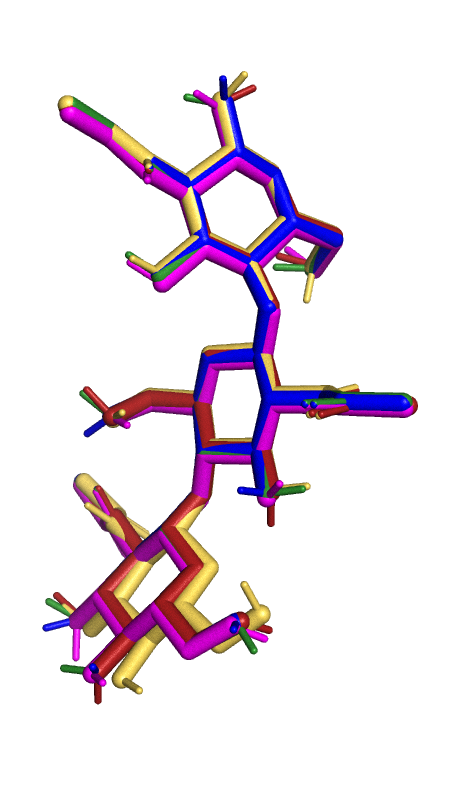
**

**Figure S2.** Superimposition of 5 runs, coloured as red, green, blue, yellow and magenta, respectively.


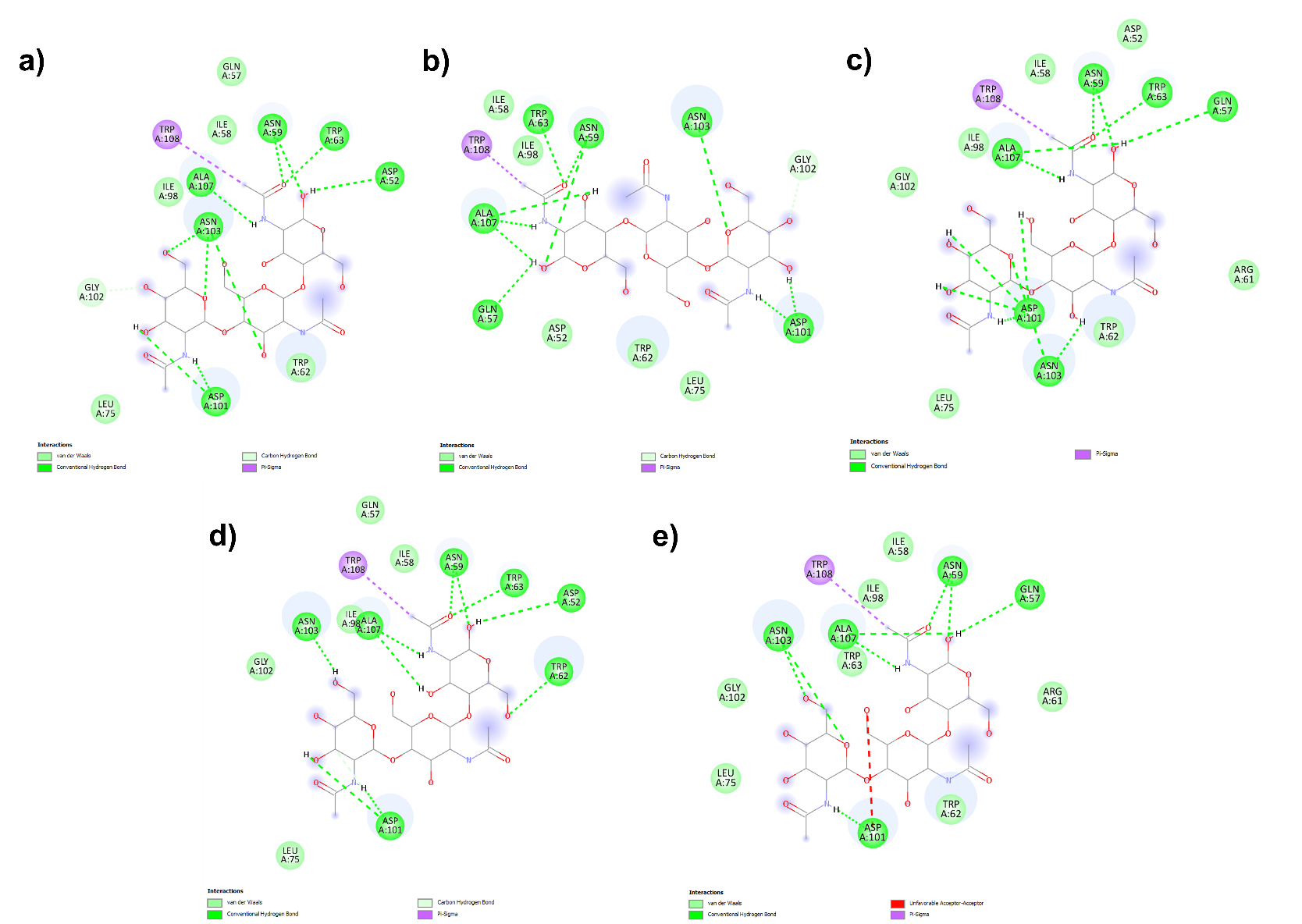


**Figure S3.** 2D interaction maps of the 5 redocking applications: a) 2D interaction map of the first redocking. b) 2D interaction map of the second redocking. c) 2D interaction map of the third redocking. d) 2D interaction map of the fourth redocking. e) 2D interaction map of the fifth redocking

**Table S1.** Table of interactions between CEWL_ZM_ and NAG_3_.

| ***Runs*** | ***H-Bond*** | ***2D Interaction Map*** |
| --- | --- | --- |
| 1 | Asp52,  Asn59,  Trp63,  Asp101,  Asn103,  Ala107 | 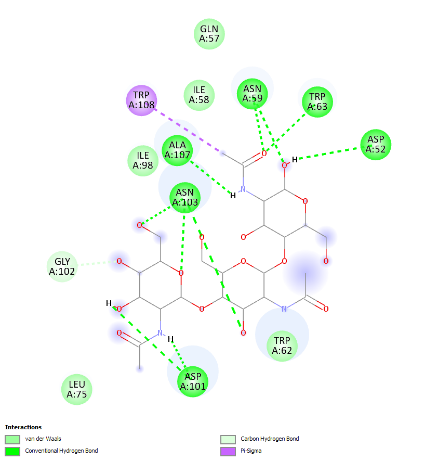 |
| 2 | Asp52,  Asn59,  Trp62,  Trp63,  Asp101,  Asn103,  Ala107 | 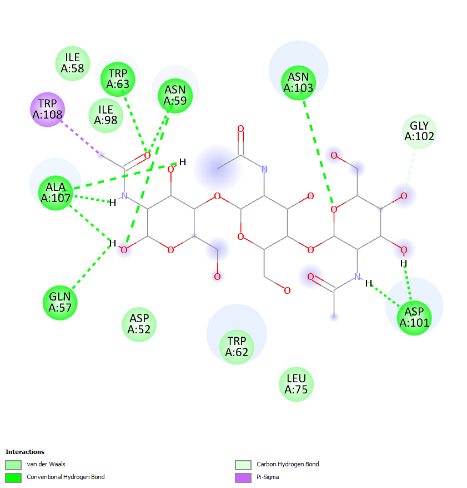 |
| 3 | Asp52,  Gln57,  Trp63,  Asp101,  Asn103,  Ala107 | 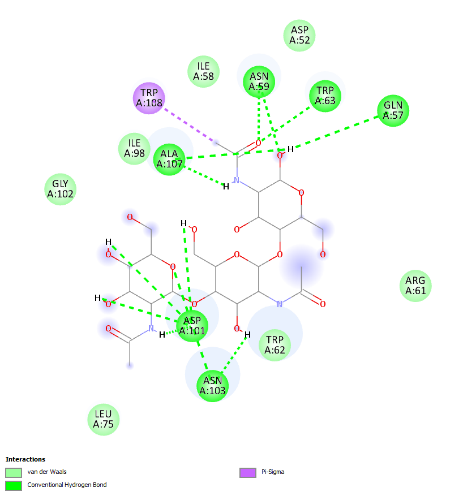 |
| 4 | Asp52,  Asn59,  Trp62,  Trp63,  Asp101,  Asn103,  Ala107 | 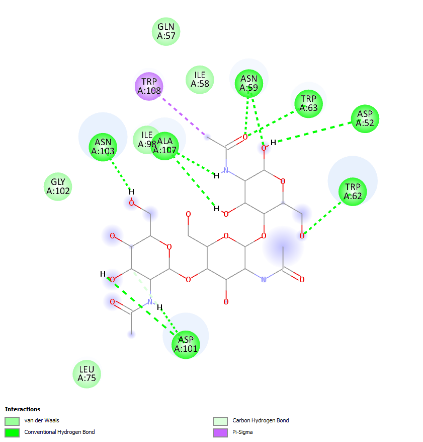 |
| 5 | Asp52,  Gln57,  Trp63,  Asp101,  Asn103,  Ala107 | 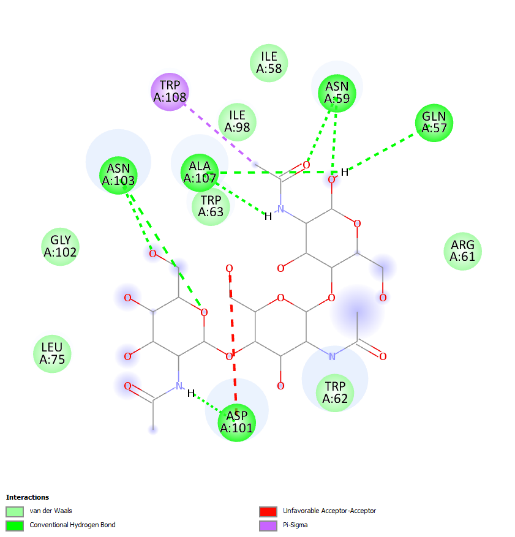 |

**Table S2.** Table of interactions between CEWL_ZM_ and TA.

| ***Runs*** | ***H-Bond*** | ***2D Interaction Map*** |
| --- | --- | --- |
| 1 | Phe34, Asn44, Asn46, Asp52, Leu56, Gln57, Arg61, Trp62, Trp63, Ile98, Asp101, Asn103, Asn106, Arg112 | 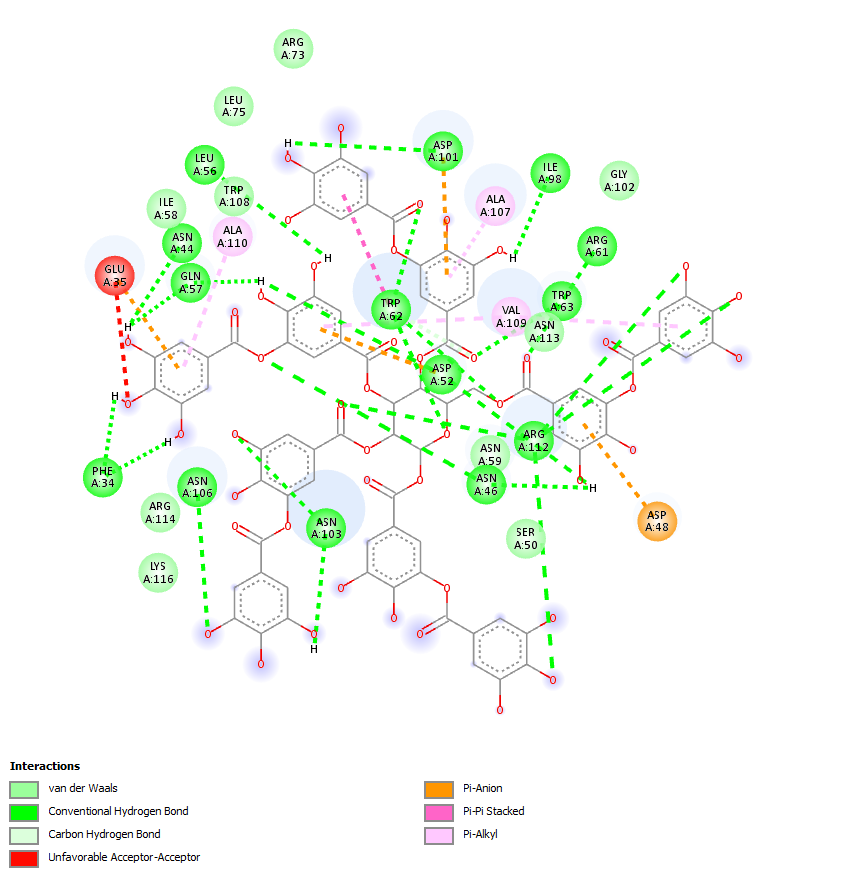 |
| 2 | Phe34, Glu35, Asn46, Asp48, Asp52, Leu56, Asn59, Trp62, Trp63, Asp101, Asn103, Asn106, Ala107, Trp108, Val109, Ala110, Arg112 | 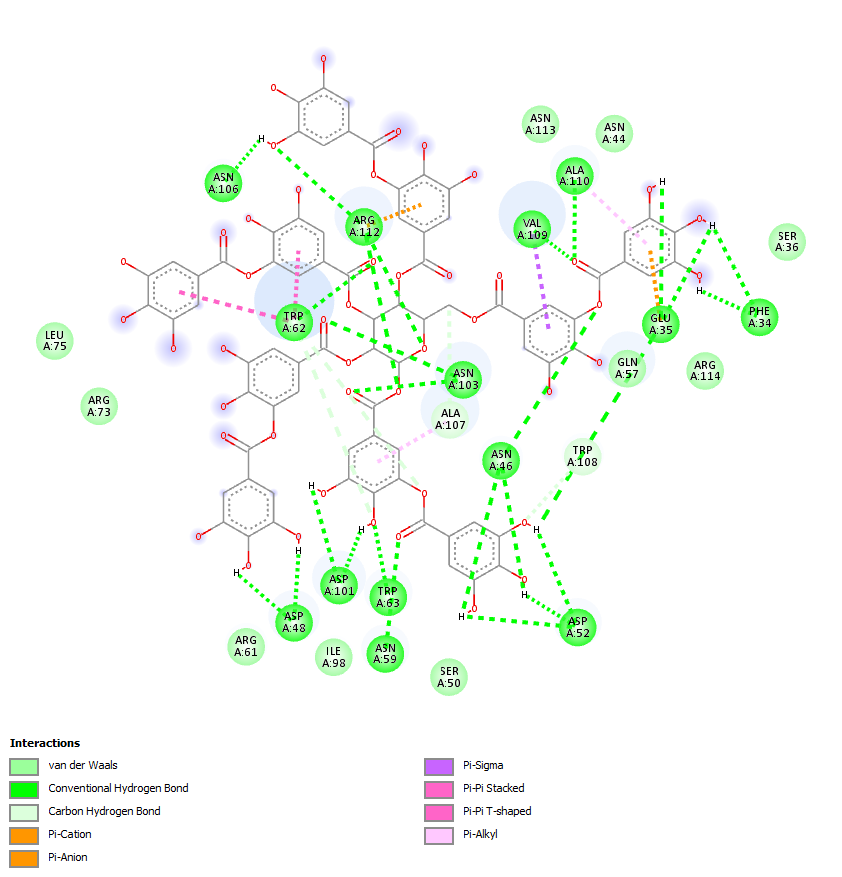 |
| 3 | Asn46, Asp48, Asp52, Leu56,  Gln57, Asn59, Trp62, Trp63,  Ile98, Asp101, Gly102, Asn103, Val109, Ala110, Arg112, Asn113, Lys116 | 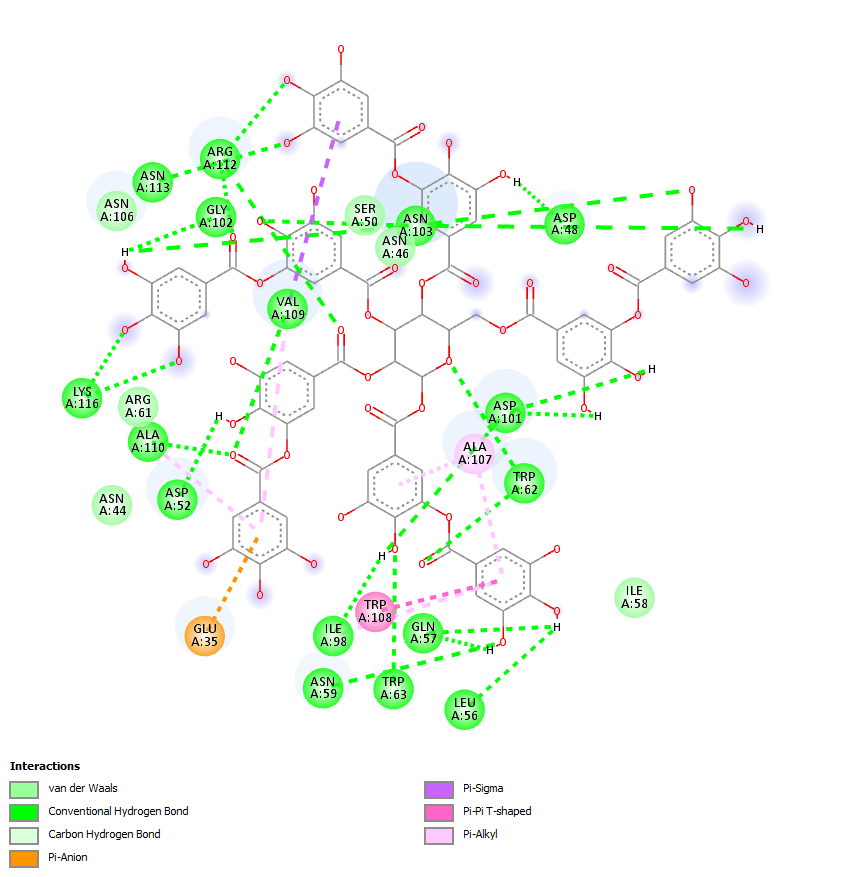 |
| 4 | Glu35, Asn44, Asn46, Asp52, Leu56,  Gln57, Trp62, Asp101, Asn103, Gly104, Ala110, Arg112,  Arg113 | 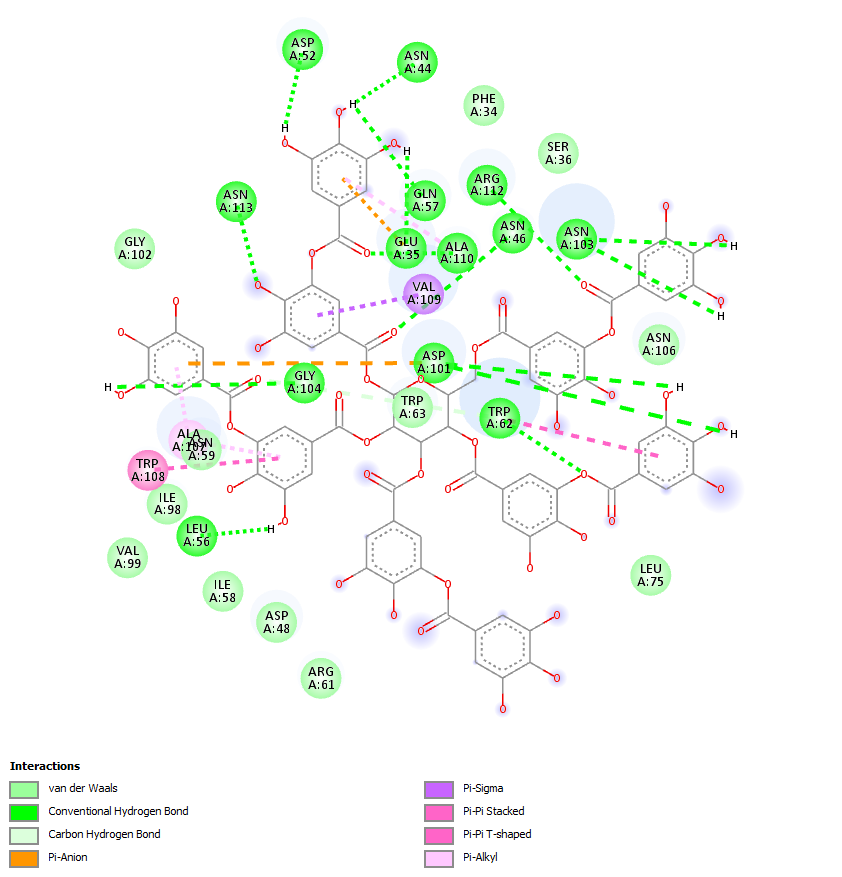 |
| 5 | Phe34, Asn44, Asn46, Asp52, Leu56, Gln57, Arg61, Trp62, Trp63, Ile98, Asp101, Asn103, Asn106, Arg112 | 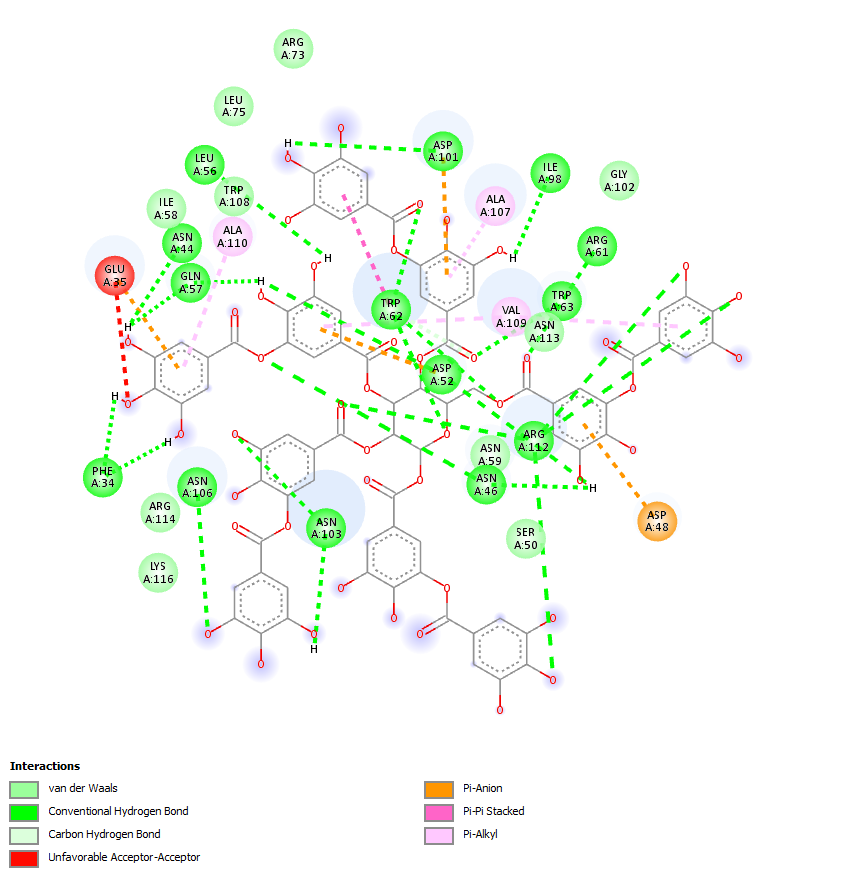 |

**
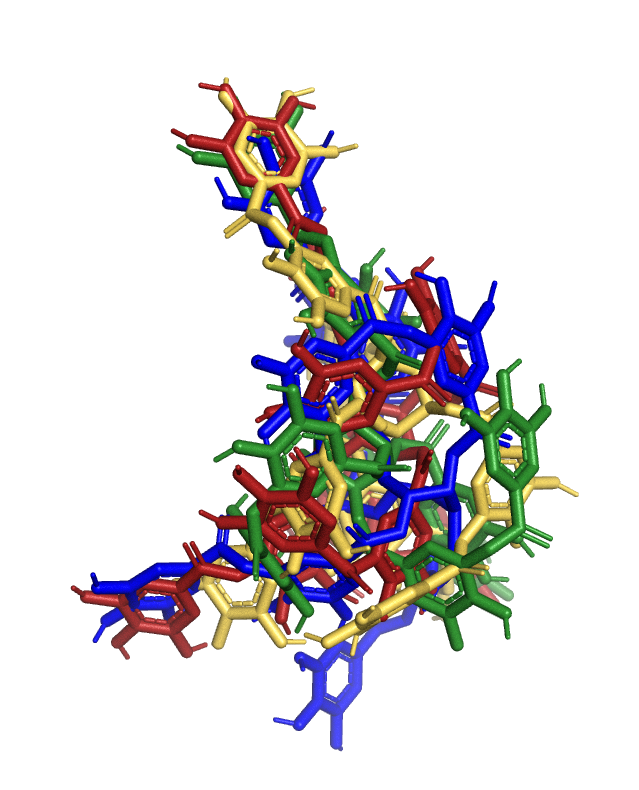
**

**Figure S4.** Superimposition of 5 runs of CEWL_ZM_-TA complex, coloured as red, green, blue, yellow and magenta, respectively.


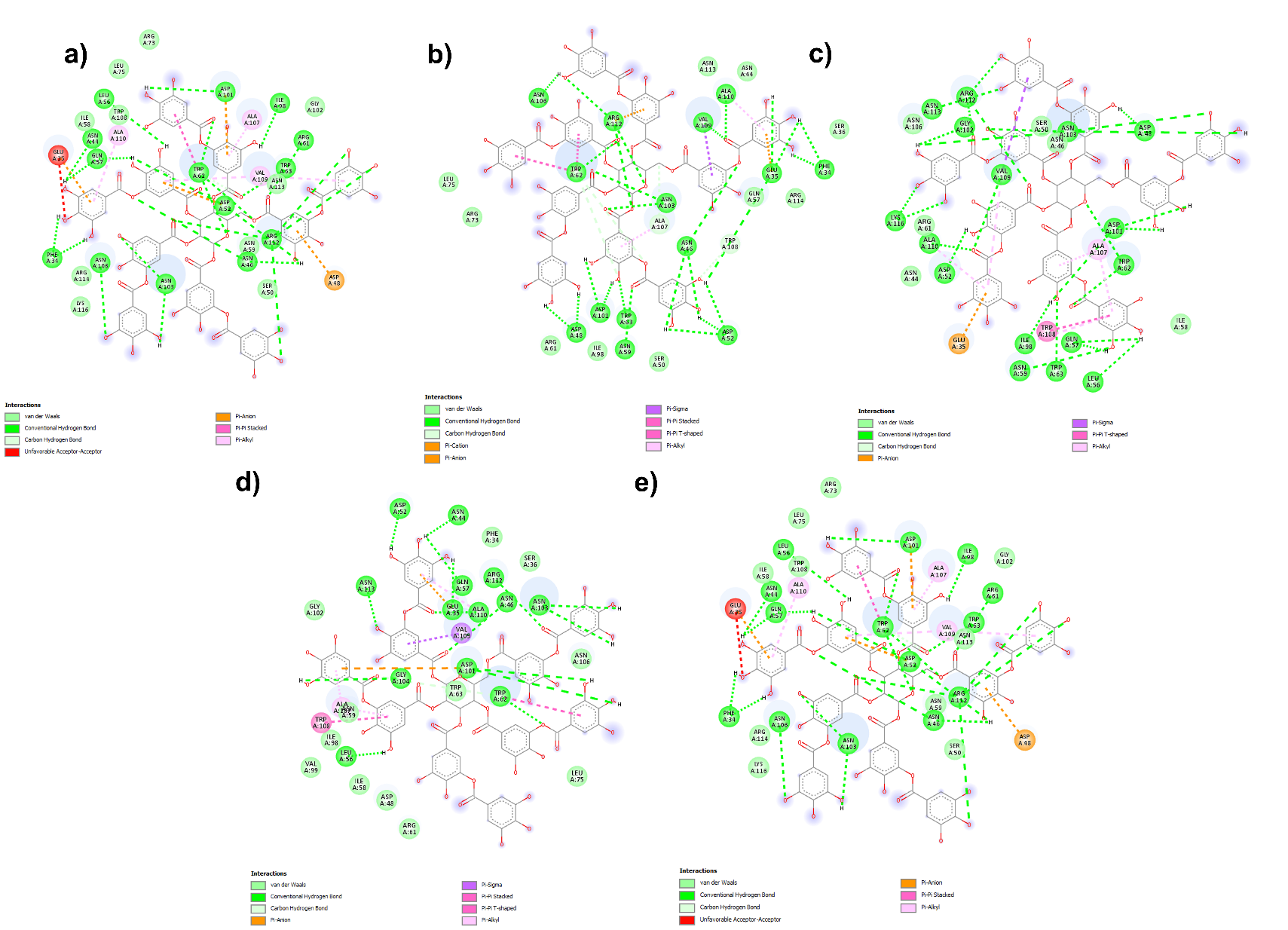


**Figure S5.** 2D interaction maps obtained from the molecular docking application for CEWL_ZM_-TA complex: a) Molecular docking run 1. b) Molecular docking run 2. c) Molecular docking run 3. d) Molecular docking run 4. e) Molecular docking run 5.


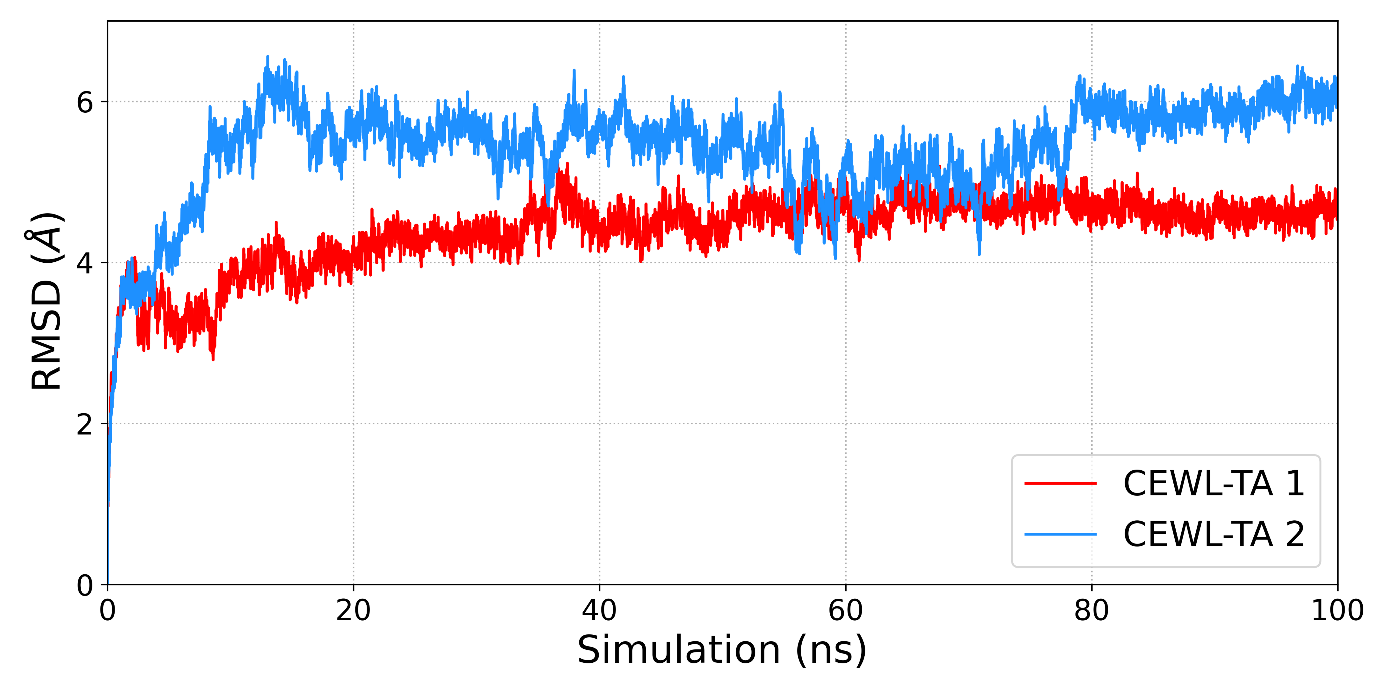


**Figure S6.** Ligand RMSD throughout the simulation.


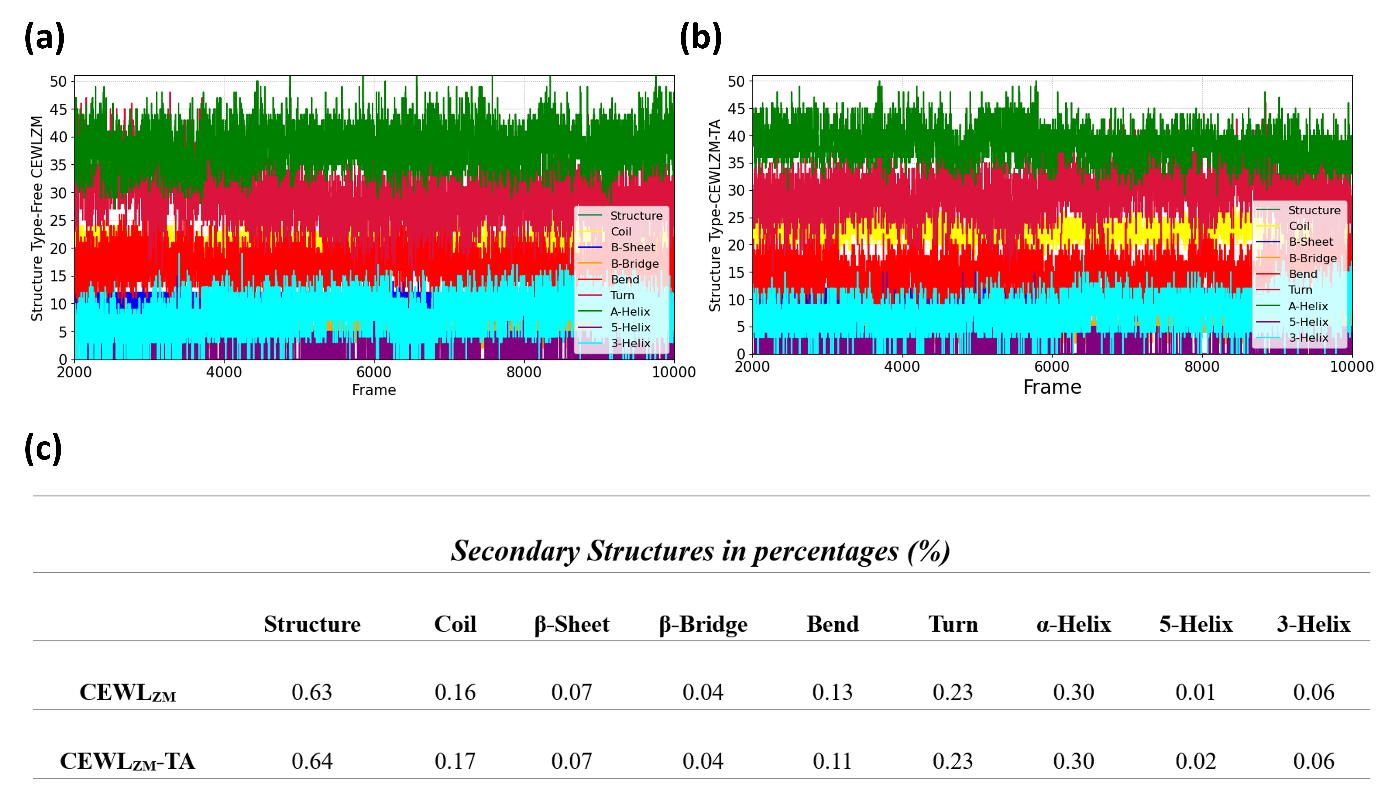


**Figure S7.** The secondary structure analysis of free and ligand-bound CEWL_ZM_. The structure components are i) structure as seagreen (indicating the sum of α-helix + β-sheet + β-bridge + turn elements), ii) coil as yellow, iii) β-sheet as blue, iv) β-bridge as orange, v) bend as red, vi) turn as crimson, vii) α-helix as green, viii) 5-helix as purple and ix) 3-helix cyan. a) The secondary structure elements of the free CEWL_ZM_. b) The secondary structure of CEWL_ZM_ in the presence of TA. c) Comparison of the secondary structure of free CEWL_ZM_ and CEWL_ZM_-TA complex.

**References**

Katsamba, P. S., Park, S., Laird-Offringa, I. A. (2002). Kinetic studies of RNA-protein interactions using surface plasmon resonance. *Methods*, *26*, 95–104. https://doi.org/10.1016/S1046-2023(02)00012-9.

Rich, R. L., Cannon, M. J., Jenkins, J., Pandian, P., Sundaram, S., Magyar, R., Brockman, J., Lambert, J., Myszka, D. G. (2008). Extracting kinetic rate constants from surface plasmon resonance array systems. *Analytical Biochemistry*, *373*, 112–120. https://doi.org/10.1016/j.ab.2007.08.017.

Cho, K. M., Deshmukh, P. R., Shin, W. G. (2021). Hydrodynamic behavior of bubbles at gas-evolving electrode in ultrasonic field during water electrolysis, *Ultrasonics Sonochemistry*, *80*, 105796. https://doi.org/10.1016/j.ultsonch.2021.105796.

Cheetham, J. C., Artymiuk, P. J., Phillips, D. C. (1992). Refinement of an enzyme complex with inhibitor bound at partial occupancy. Hen egg-white lysozyme and tri-N-acetylchitotriose at 1.75 Å resolution, *Journal of Molecular Biology*, *224*, 613–628. https://doi.org/10.1016/0022-2836(92)90548-X.
